# Dupilumab and cutaneous T-cell lymphoma: A retrospective cohort study

**DOI:** 10.1016/j.jdin.2026.02.004

**Published:** 2026-02-23

**Authors:** Sarah Baghdasarian, Ohoud Al-Saedi, Raphael E. Cuomo

**Affiliations:** University of California, San Diego, School of Medicine, San Diego, California

**Keywords:** alopecia, biologic therapy, cutaneous T-cell lymphoma, dupilumab, herpes, urticaria

*To the Editor:* Dupilumab is a monoclonal antibody that blocks interleukin 4 and interleukin 13 receptor signaling.^1^ First approved in 2017 for atopic dermatitis, it now has approvals across several dermatologic diseases, including prurigo nodularis, chronic spontaneous urticaria, and bullous pemphigoid.^2^ Recent reports raise concern for rare adverse events, including cutaneous T-cell lymphoma, alopecia, herpes simplex virus reactivation, and urticaria.^3^, ^4^, ^5^

We performed a retrospective cohort study using electronic health records from the University of California San Diego Health system under IRB exemption 1604619-1. We identified all patients who received dupilumab in routine care after FDA approval, without restriction on age, dose, indication, or route (Table I. Most initiations were for atopic dermatitis, with other indications uncommon. Cutaneous T-cell lymphoma outcomes were defined by diagnosis codes for mycosis fungoides or Sézary syndrome recorded at least 3 months after dupilumab initiation. The comparison group comprised system patients without dupilumab exposure and without cutaneous T-cell lymphoma. Records were queried on secure servers and not exported.

**Table I:** Demographic characteristics for analytic comparison groups

	Overall	Post-dupilumab CTCL+	Post-dupilumab CTCL−
Overall, *n* (%)	1374	3 (0.2)	1371 (99.8)
Age, mean ± SD	55 ± 11	45 ± 2	56 ± 19
Race, *n* (%)			
White	762 (55.5)	2 (0.3)	760 (99.7)
Asian	224 (16.3)	1 (0.4)	223 (99.6)
Black or African American	67 (4.9)	0 (0)	67 (100.0)
Multirace	31 (2.3)	0 (0)	31 (100.0)
Native Hawaiian or Other Pacific Islander	10 (0.7)	0 (0)	10 (100.0)
Other race	201 (14.6)	0 (0)	201 (100.0)
Unknown	72 (5.2)	0 (0)	72 (100.0)
American Indian or Alaska Native	7 (0.5)	0 (0)	7 (100.0)
Gender, *n* (%)			
Male	630 (45.9)	3 (0.5)	627 (99.5)
Female	744 (54.1)	0 (0)	744 (100.0)

*CTCL*, Cutaneous T-cell lymphoma.

Prespecified safety outcomes were cutaneous T-cell lymphoma, alopecia, herpes simplex virus reactivation, and urticaria, selected a priori based on published safety signals and pharmacovigilance hypotheses. Absolute risks were calculated for dupilumab users and nonusers. Multivariable logistic regression estimated associations adjusting for age, sex, race, and baseline atopic dermatitis at the index encounter. To reduce bias from prodromal presentation and surveillance intensity, the model also included 4 encounter context covariates that were finalized before modeling: nonspecific rash or eruption and pain as proxies for early indeterminate presentations, prior cutaneous squamous cell carcinoma as a proxy for dermatology contact, and an internal workflow flag recorded as therapeutic drug monitoring that indicates protocolized follow up rather than a serum assay.

The cohort included 1374 dupilumab treated patients and 1,249,409 controls. Three dupilumab recipients developed cutaneous T-cell lymphoma at least 3 months after initiation, with a mean time to diagnosis of 266.3 ± 173.1 days. Absolute risks among dupilumab users versus non-users were 0.22 percent versus 0.042 percent for cutaneous T-cell lymphoma, 2.84 percent versus 0.87 percent for alopecia, 4.00 percent versus 1.61 percent for herpes simplex virus reactivation, and 5.24 percent versus 0.65 percent for urticaria. In the adjusted model, dupilumab exposure remained associated with higher odds of cutaneous T-cell lymphoma with covariate effects shown in Table II. Secondary outcomes were analyzed in parallel with identical adjustment to contextualize the broader safety profile.

**Table II:** The multivariable logistic model for cutaneous T-cell lymphoma with dupilumab exposure adjusted for age, sex, race, baseline atopic dermatitis, and 4 encounter-context covariates

Covariate	Odds ratio	Error	*P* value
Intercept	2.696 × 10^14^	5.287	<.001
Dupilumab user	1.872	0.279	.025
Diagnosis: eczema	1.935	0.425	.120
Diagnosis: pain	0.451	0.200	<.001
Diagnosis: rash	0.678	0.120	.001
Diagnosis: squamous cell	1.338	0.367	.428
Diagnosis: therapeutic drug monitoring	0.660	0.133	.002
Year of birth	0.981	0.003	<.001
Race: Asian	0.607	0.197	.011
Race: Black	1.669	0.195	.009
Race: Multi	0.416	0.505	.086
Race: Native	6.306 × 10^−4^	207.436	.954
Race: other	1.387	0.123	.008
Race: Pacific Islander	0.520	1.004	.516
Race: unknown	0.655	0.415	.307
Gender: female	0.551	0.099	<.001

Nonspecific rash or eruption and pain capture likely prodromal CTCL presentations that are commonly miscoded at treatment initiation. Prior cutaneous squamous cell carcinoma and the internal workflow flag labeled therapeutic drug monitoring index dermatology contact intensity and protocolized follow up; the flag is a scheduling indicator, not a laboratory assay. These variables are included to reduce confounding from baseline misclassification and surveillance bias.

Potential mechanisms for a lymphoma signal include unmasking of early disease in refractory dermatitis and altered interleukin 13 pathways supporting malignant T-cell proliferation. Clinically, we recommend thorough baseline skin examination with photographic documentation, a low threshold for biopsy in atypical or treatment refractory dermatitis, and interval reassessment during dupilumab therapy.

Limitations include small lymphoma counts, single center design, limited histopathology detail, and possible misclassification of indication. Larger multicenter longitudinal studies with pathology confirmation are needed.

In conclusion, within a large health system cohort, dupilumab use was associated with rare but increased risk of cutaneous T-cell lymphoma and several other dermatologic adverse events. Vigilant baseline assessment and ongoing monitoring are warranted when initiating dupilumab.

## Conflicts of interest

None disclosed.

## References

[1] Simpson E.L., Bieber T., Guttman-Yassky E., SOLO 1 and SOLO 2 Investigators (2016). Two phase 3 trials of dupilumab versus placebo in atopic dermatitis. N Engl J Med.

[2] Sanofi (2024). Dupixent® (dupilumab) U.S. label updated with data further supporting use in atopic dermatitis with moderate-to-severe hand and foot involvement. https://www.sanofi.com/en/media-room/press-releases/2024/2024-01-16-12-00-00-2809681.

[3] Hasan I., Parsons L., Duran S., Zinn Z. (2024). Dupilumab therapy for atopic dermatitis is associated with increased risk of cutaneous T cell lymphoma: a retrospective cohort study. J Am Acad Dermatol.

[4] Jfri A., Smith J.S., Larocca C. (2023). Diagnosis of mycosis fungoides or Sézary syndrome after dupilumab use: a systematic review. J Am Acad Dermatol.

[5] Mandel J., Mehta J., Hafer R. (2024). Increased risk of cutaneous T-cell lymphoma development after dupilumab use for atopic dermatitis. Dermatol Ther.

